# The future of pharmacology and therapeutics of the arachidonic acid cascade in the next decade: Innovative advancements in drug repurposing

**DOI:** 10.3389/fphar.2024.1472396

**Published:** 2024-08-29

**Authors:** Paola Patrignani, Annalisa Contursi, Stefania Tacconelli, Dieter Steinhilber

**Affiliations:** ^1^ Systems Pharmacology and Translational Therapeutics Laboratory, The Center for Advanced Studies and Technology (CAST), “G. d’ Annunzio” University, Chieti, Italy; ^2^ Department of Neuroscience, Imaging and Clinical Science, “G. d Annunzio” University Medical School, Chieti, Italy; ^3^ Institute of Pharmaceutical Chemistry, Goethe University Frankfurt, Frankfurt, Germany

**Keywords:** drug repurposing, antiplatelet agents, systems biology and network analysis, 5-lipoxygenase, cancer

## Abstract

Many drugs can act on multiple targets or disease pathways, regardless of their original purpose. Drug repurposing involves reevaluating existing compounds for new medical uses. This can include repositioning approved drugs, redeveloping unapproved drugs, or repurposing any chemical, nutraceutical, or biotherapeutic product for new applications. Traditional drug development is slow, expensive, and has high failure rates. Drug repurposing can speed up the process, costing less and saving time. This approach can save 6–7 years of early-stage research time. Drug repurposing benefits from existing compounds with optimized structures and approved for clinical use with associated structure-activity relationship publications, supporting the development of new effective compounds. Drug repurposes can now utilize advanced *in silico* screening enabled by artificial intelligence (AI) and sophisticated tissue and organ-level *in vitro* models. These models more accurately replicate human physiology and improve the selection of existing drugs for further pre-clinical testing and, eventually, clinical trials for new indications. This mini-review discusses some examples of drug repurposing and novel strategies for further development of compounds for targets of the arachidonic acid cascade. In particular, we will delve into the prospect of repurposing antiplatelet agents for cancer prevention and addressing the emerging noncanonical functionalities of 5-lipoxygenase, potentially for leukemia therapy.

## 1 Introduction

The future of pharmacology and therapeutics in the next decade depends on several crucial factors, such as the discovery of new drug targets, the evolution of drug delivery systems, and the refinement of precision tools for personalized medicine. A debate centers around whether we should focus on developing new drugs to treat human diseases or if it is better to repurpose existing compounds by enhancing our understanding of their mechanisms of action using new tools such as omics technology and artificial intelligence. It is important to note that current cancer treatments have significant toxicity and can significantly impact patients’ lives, even if they provide a few extra years of life. This underscores the need for effective drug delivery systems and personalized patient treatment. However, our current tools for characterizing patients’ clinical backgrounds and individual responses to treatments still need to be optimized. This mini-review focuses on effective strategies for reducing the time and cost of drug development by drug repurposing (or repositioning) for targets of the arachidonic acid cascade, which allows the identification of new therapeutic applications of already approved drugs. We will explore specific examples of drug repurposing and innovative strategies for further advancement in the field. Thus, we focus on the potential use of antiplatelet agents for cancer prevention and examine the discovery of noncanonical roles of 5-lipoxygenase (5-LO), particularly in the context of therapy for leukemia.

## 2 Repurposing antiplatelet agents in cancer prevention

De Angelis et al. (2024) recently reported that in 2020, 23.7 million people in Europe (12.8 million women and 10.9 million men) had been diagnosed with cancer at some point in their lives, either recently or in the past. This accounts for 5% of the population, which is expected to increase as the population ages. The high cost of innovative therapies and an aging population are challenging the sustainability of healthcare and social systems. Therefore, it is crucial to prioritize primary prevention and early diagnosis to reduce the number of patients and improve the chances of recovery.

A large-scale study carried out in the United States found that removing colorectal adenoma as a precancerous lesion can reduce the risk of death from colorectal cancer by 53% (Zauber et al., 2012). Therefore, endoscopic resection is performed globally for the treatment of colorectal adenomas. For pre-colectomy and post-colectomy patients, chemoprevention aims to prevent or slow down the growth of adenomas. This can lower the risk of cancer development, thus reducing the need for surgery and delaying the need for surveillance endoscopy. Adding low-dose aspirin can be an effective and cost-efficient strategy because it prevents proximal colorectal adenomas (Hassan et al., 2012).

**FIGURE 1:**
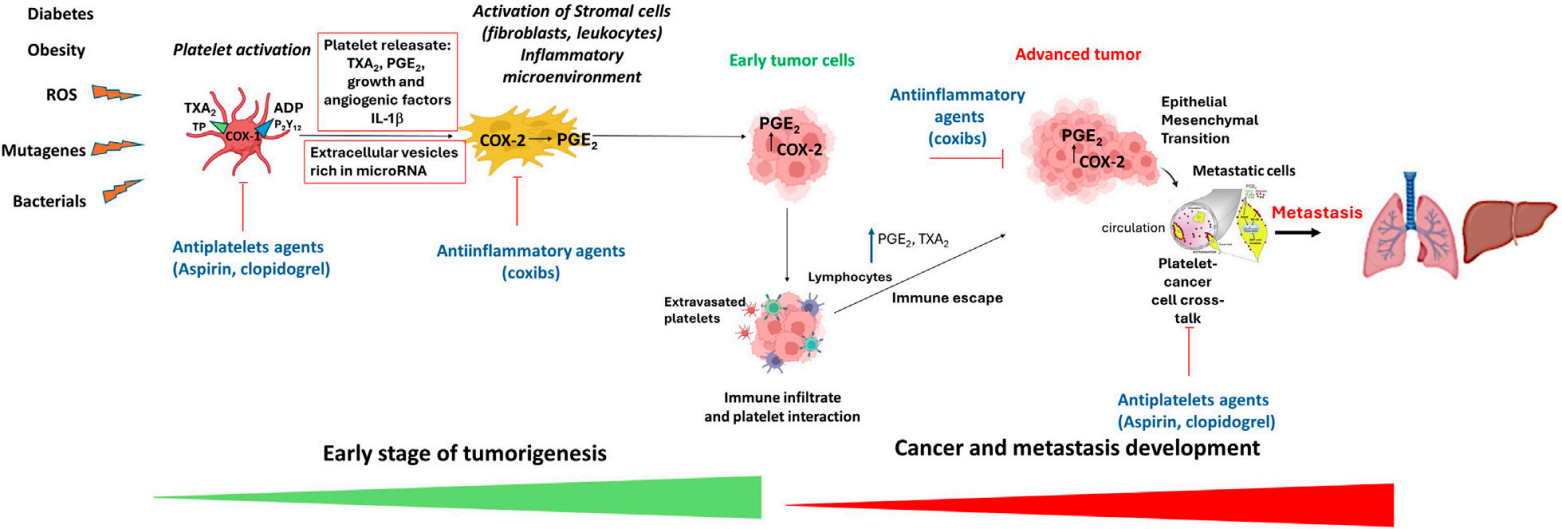
The platelet hypothesis of tumorigenesis and mechanisms of antitumor effects of antiplatelet agents and anti-inflammatory drugs. In the early stages of tumor formation, activated platelets release growth and angiogenic factors, thromboxane (TX) A_2_ and adenosine diphosphate (ADP), which contribute to the development of an inflammatory microenvironment via the induction of COX-2 (cyclooxygenase-2) expression leading to aberrant generation to prostaglandin (PG) E_2_. PGE_2_ is a central mediator in inflammation and contributes to acquiring tumor properties, such as proliferation, inhibition of apoptosis, migration, and immune escape. These processes promote the growth and survival of early tumor cells. Tumor progression is associated with immune infiltration, extravasated platelets, and enhanced PGE_2_ and TXA_2_ generation that contribute to immune escape. In advanced tumors, COX-2-dependent PGE_2_ contributes to epithelial-mesenchymal transition (EMT). In the bloodstream, platelets interact with cancer cells, enabling them to spread and colonize other tissues through various mechanisms, including EMT. Antiplatelet agents like aspirin and clopidogrel, by inhibiting platelet activation, prevent the induction of COX-2 and PGE_2_, thus affecting early tumor development. Moreover, antiplatelet agents constrain tumor progression and metastasis. Anti-inflammatory drugs, such as coxibs (selective COX-2 inhibitors), can impede cancer development, progression and metastasis by inhibiting the activity of COX-2.

There is compelling evidence that low-dose aspirin could inhibit the initial stages of colorectal adenoma development, resulting in long-term colorectal adenoma and cancer prevention (Patrignani and Patrono, 2016). Low-dose aspirin is an antiplatelet drug that mainly affects the biosynthesis of platelet thromboxane (TX)A_2_ by irreversibly affecting the activity of cyclooxygenase (COX)-1. TXA_2_ has biologically relevant roles in hemostasis (including platelet aggregation), vascular tone (contraction of vascular smooth muscle cells, VSMC), cell proliferation, and migration. TXA_2_ regulates the tumor microenvironment by modulating angiogenic potential, tumor extracellular matrix (ECM) stiffness, and host immune response (Ashton et al., 2022). Thus, it has been proposed that aspirin can limit the development of colorectal tumors by affecting platelet-dependent signaling pathways triggered during the early stages of colorectal adenomatous lesion development (Patrignani and Patrono, 2016; 2018) (Figure 1). Our recent findings support this hypothesis (Bruno et al., 2022). *Apc* ^*Min/+*^ mice (an animal model bearing multiple intestinal neoplasia) with the specific deletion of Ptgs1 (protein name COX-1) in the megakaryocytes/platelets (mimicking the pharmacodynamics of low-dose aspirin in humans) were associated with a reduced number (67%) and size (29%) of tumors of the small intestine (Bruno et al., 2022). One of the first events in developing intestinal adenomatous lesions is the induction of COX-2 in myofibroblasts, leading to enhanced biosynthesis of prostaglandin (PG)E_2_, which is a crucial pathway in cancer as it prevents apoptosis while promoting migration, proliferation, angiogenesis, and immune evasion (Wang and Dubois, 2010; 2014). Extravasated platelets are detected in conjunction with myofibroblasts in the inflamed colon (Sacco et al., 2019) and in intestine tissue sections of *Apc* ^*Min/+*^mice (Bruno et al., 2022). The release of platelet TXA_2_ activates myofibroblast TXA_2_ receptors (TP) involved in COX-2 expression, enhances proliferative and migratory abilities, and expression of mesenchymal markers such as vimentin in myofibroblasts (Bruno et al., 2022; Sacco et al., 2019).

Aspirin can indirectly prevent the upregulation of COX-2-dependent signaling pathways that promote cancer development by affecting platelet function (Patrignani and Patrono, 2016; 2018). Rodríguez-Miguel et al. (2019) performed an epidemiologic study on the association between Clopidogrel intake, another antiplatelet agent acting by preventing the activation of the P2Y12 receptor for ADP, alone or associated with aspirin, and colorectal cancer (CRC) incidence using a large population-based database of electronic health records in Spain. The authors found that clopidogrel intake has a similar effect on CRC as low-dose aspirin. However, the mechanism of these protective effects remains to be elucidated. Notably, the possible improved cancer prevention by the coadministration of low-dose aspirin and a P2Y12 antagonist (dual antiplatelet therapy) remains to be clarified. Clopidogrel, one of the ADP receptor antagonist family, is recommended for the reduction of atherosclerotic events, including myocardial infarction, ischemic stroke, and vascular death in patients with atherosclerosis manifested by recent stroke, myocardial infarction, or established peripheral vascular disease (Jarvis and Simpson, 2000). Clopidogrel is available as a generic medication. Retrospective database analysis has shown that generic clopidogrel is comparable to its brand-name counterpart regarding cardiovascular and bleeding outcomes for treating patients with acute myocardial infarction (Chan et al., 2023). Both low-dose aspirin and clopidogrel used as monotherapy enhance, to a comparable extent, the risk of upper gastrointestinal bleeding (García Rodríguez et al., 2011). However, the risk of upper gastrointestinal bleeding is relatively low for individuals under 70 years of age.

ADP is an important platelet agonist that is actively secreted from platelet-dense granules but is also passively released from damaged erythrocytes, endothelial cells, and apoptotic cancer cells (Woulfe et al., 2001). Along with TXA_2_, it amplifies platelet functions, and there is an important interplay between the two in promoting platelet activation in response to low concentrations of primary agonists such as collagen and thrombin (Tacconelli et al., 2020). Platelets store a significant amount of proteins in α-granules, including angiogenic, inflammatory, and growth factors released upon activation by specific signaling pathways (Quinton et al., 2004). ADP-induced α-granule release through costimulation of G_αq_ and G_αi_ signaling. The TXA_2_ receptor, which signals via G_α12/13_ and G_αq_, requires the P2Y_12_ receptor G_αi_ signal for α-granule release. Hence, blocking the P2Y_12_ receptor is more effective than aspirin in inhibiting protein release from platelet α-granules. We have discovered that when platelets and cancer cells are cultured together, inhibiting platelet COX-1 using aspirin or blocking the P2Y_12_ receptor with antagonists can prevent the platelet-induced epithelial-mesenchymal transition (EMT) phenomenon and migration of cancer cells *in vitro* (Guillem-Llobat et al., 2016). These events are critical factors in cancer metastasis. This response is associated with inhibition of platelet activation and reduced generation of TXA_2_ and PGE_2_, which are involved in EMT. Furthermore, our research has revealed that extracellular vesicles (EVs) derived from platelets of CRC patients are implicated in acquiring prothrombotic phenotypes in cancer cells (Contursi et al., 2023). The increase in the generation of TXA_2_ in cancer cells mediates this process. In addition, bound platelets or platelet aggregates may enhance the survival of circulating tumor cells by protecting them from immune destruction or physical damage caused by shear stress in the microcirculation. As a result, curbing or inhibiting platelet activation in cancer patients may mitigate metastasis, and individuals at risk of cancer-associated thrombosis might benefit from that treatment (Wright et al., 2020).

Of note, aspirin and clopidogrel are both irreversibly acting drugs. Aspirin acetylates both COX isoforms at a serine residue in the active site (Roth et al., 1975), whereas clopidogrel forms a disulfide bridge with extracellular cysteines of the P2Y_12_ receptor (Savi et al., 2000; Ding et al., 2003). In contrast to noncovalent binding mechanisms, this mode of action requires *de novo* synthesis of the target protein to restore protein activity, and the duration of drug action usually mainly depends on the half-life of the target protein in the respective cell. In platelets, there is limited *de novo* protein biosynthesis, so there is a sustained action of irreversibly binding drugs, which correlates with the lifespan of platelets of around 10 days. Thus, irreversible inhibitors are very effective in the inhibition of platelet-derived proteins. In the case of aspirin, this leads to sustained and effective inhibition of TXA_2_ formation in platelets, whereas prostaglandin formation in other cell types is less affected by low-dose aspirin. Some clinical trials are ongoing and are assessing the impact of low-dose aspirin in cancer prevention, such as CAPP3 in Lynch syndrome (https://www.capp3.org/) and the Add aspirin trial in four types of cancer (colorectal, breast, prostate, and esophageal cancer) (Coyle et al., 2016). New trials will be designed to assess the impact of clopidogrel and other P2Y_12_ inhibitors in adenoma prevention in patients with sporadic cancer. The importance of dual antiaggregatory inhibition by the two drugs to improve anticancer efficacy remains to be investigated.

## 3 Developing a systems biology approach and network analysis to explore deregulated biological processes and identify potential drug-repurposing candidates

Network-based analysis is an established method for *in silico* examination of the intricate nature of biological systems and assessing interactions among the various participants. This approach serves as a robust instrument for correlating pharmacological and disease data. Recent systems biology methodologies, leveraging network analysis, have explored novel applications for existing drugs, anticipated potential new anticancer therapeutic avenues, and pinpointed promising targets for further investigation. Here, we report some examples of this approach.

The biological response to disease, inflammation, is critical to many pathological states. Managing inflammation with drugs is crucial in medical practice. Anti-inflammatory drugs work by targeting specific molecules involved in the inflammatory response. These drugs are typically classified as steroidal and non-steroidal. However, their effects often extend beyond their intended targets, impacting other molecules and biological functions, which can result in secondary therapeutic applications or adverse drug reactions (ADRs). Using network models, a recent study (de Anda-Jáuregui et al., 2018) explored relationships among anti-inflammatory drugs, functional pathways, and ADRs. The study used network-based properties such as degree, clustering coefficient, and node strength to identify new therapeutic applications and ADR risks for these drugs. These parameters have identified naproxen, meloxicam, etodolac, tenoxicam, flufenamic acid, fenoprofen, and nabumetone as potential candidates for drug repurposing due to their lower ADR risk. This network-based analysis offers a new approach to examining the impact of drugs in a therapeutic setting.

Metabolic syndrome is a complex condition characterized by the combined occurrence of obesity, high blood sugar levels, elevated blood pressure, increased concentrations of triglycerides, and decreased levels of high-density lipoprotein cholesterol. A systems biology approach employing network analysis has been utilized to investigate the biological processes associated with the syndrome and explore potential drug treatments (Misselbeck et al., 2019). The approach suggests that the Bruton Tyrosine Kinase (BTK) inhibitor ibrutinib could be a novel pharmacological treatment, as it effectively lowers inflammation in an obesity model. BTK inhibitors impede the activity of the enzyme BTK, a critical component within the B-cell receptor signaling pathway. Specific B-cell leukemias and lymphomas rely on B-cell receptor signaling to promote their growth and survival.

## 4 5-Lipoxygenase and cancer

The 5-LO pathway was discovered in the seventies of the last century (Borgeat et al., 1976). It was observed that arachidonic acid (AA) is oxidized at carbon C(5), which finally leads to the generation of leukotrienes (LT) where 5-LO acts in concert with the down-stream leukotriene synthases LTA_4_ hydrolase and LTC_4_ synthase (Samuelsson, 1987). Studies on the biological roles of these lipid mediators have uncovered that LTs are mediators of inflammatory and allergic responses, that they play a role in the innate immune system, and are involved in host defense reactions (Peters-Golden and Henderson, 2007; Flamand et al., 2007; Serenzani et al., 2023). Due to the pro-inflammatory functions of LTs and other 5-LO-derived oxylipins, 5-LO became an interesting target for inhibitor development. However, none of the 5-LO and FLAP inhibitors reached the market except for zileuton. One possible explanation is that the sole inhibition of 5-LO and LT formation is not effective enough to treat inflammatory diseases (Sala et al., 2018). Apart from its role in inflammation, the involvement of 5-LO in cancer development has been suggested by many studies in different tissues (for review, see Steinhilber et al., 2010). For BCR-ABL-positive leukemia in mice, it was shown that knockout of 5-LO prevents CML induction, which seems to be due to a defect in leukemic stem cell proliferation and long-term survival in the absence of 5-LO (Chen et al., 2009). Treatment with high doses of the 5-LO inhibitor zileuton had the same effect (Chen et al., 2009). In line with this, we could show that pharmacological inhibition of 5-LO by CJ-13,610 also affects the aberrant stem cell capacity in a PML/RARα-positive model of AML (acute myeloid leukemia) and a murine cancer stem cell (CSC) model (Roos et al., 2014). The specific mechanisms accountable for these effects are not wholly understood. One observation was that certain 5-LO inhibitors prevent the translocation of beta-catenin into the nucleus and thus inhibit Wnt-signalling, which regulates the stemness of myeloid progenitor cells (Roos et al., 2014). The interaction of 5-LO with beta-catenin is supported by proximity ligation assays, which demonstrated the cellular co-localization of 5-LO and beta-catenin and the inhibition of beta-catenin translocation into the nucleus with the 5-LO inhibitors (Brand et al., 2018; Roos et al., 2014). Although the mode of interaction between 5-LO and beta-catenin is unclear at the moment, the inhibitor experiments and the proximity ligation assays suggest that both proteins might be located in the same protein complex. Of note, depending on its role in cellular responses, 5-LO can be localized at different intracellular sites. In resting cells, the protein can be present in the cytosol or the nucleus within euchromatin, whereas inflammatory stimuli lead to the translocation of 5-LO to the nuclear membrane, where it interacts with FLAP for LT formation. Recently, it was shown that the nuclear localization of 5-LO in leukocytes is related to a noncanonical function as a regulator of gene expression, which seems to be independent of the classical enzymatic activity (Kreiß et al., 2022). Among many others, COX-2 and kynureninase (i.e., an enzyme responsible for the formation of 3-OH-anthranilic acid) were identified as direct 5-LO target genes, and pathway analyses revealed that many genes involved in the regulation of inflammatory responses, cell adhesion, and cell proliferation are regulated by 5-LO. The interaction of 5-LO with dicer is another noncanonical function of the 5-LO protein (Provost et al., 1999). Recently, we have shown that 5-LO induces the expression of the miR-99b/let-7e/125a cluster at the pri-miR level but inhibits the processing of pre-let-7e by dicer so that 5-LO upregulates expression of the mature miR-99b as well as miR-125a whereas the cellular let-7e levels are kept constant (Uebbing et al., 2021). This is particularly interesting as these miRNAs regulate myeloid cell differentiation and play an important role in tumorigenesis and developmental processes. Thus, apart from its key role in oxylipin formation, 5-LO has several noncanonical functions which seem to be at least partially independent of its classical enzymatic activity and which are not affected by the classical 5-LO inhibitors (Kahnt et al., 2024). Thus, it might be interesting to target these noncanonical 5-LO functions by developing new inhibitors and, therefore, to address cell adhesion and developmental processes, which might be helpful for leukemia treatment. Since 5-LO is mainly expressed in leukocytes, this approach would allow for the specific interference of signaling processes in these blood cell types.

## 5 Conclusion

Drug repurposing, also called repositioning or reprofiling, identifies new uses for approved or investigational drugs. This can result from accidental discoveries of off-target effects or newly recognized actions. For example, thalidomide, withdrawn from the market due to birth defects, was later repurposed to treat leprosy and multiple myeloma. During the HIV/AIDS crisis, the drug azidothymidine (AZT), originally developed to treat certain cancers, was repurposed as a potent anti-HIV compound. This effort quickly advanced from lab testing to clinical use, with FDA approval in 1987 for managing HIV. During the COVID-19 pandemic, drug repurposing has progressed rapidly, with over 100 drugs moved into clinical trials for COVID-19 between January 2020 and December 2021. Four drugs have received FDA approval or emergency use authorization, and an additional 15 drugs have been recommended for off-label use. This approach enables faster development during emergencies, reduces safety risks and costs, and could lead to discovering new targets for novel therapeutics. The potential of drug repurposing for commercial use has been hindered by patent and market challenges. However, there is increasing interest from clinicians, foundations, and government agencies, driven by the need for quick patient solutions and preparedness for pandemics. Drug repurposes can now utilize advanced *in silico* screening enabled by AI and sophisticated tissue and organ-level *in vitro* models. These models more accurately replicate human physiology, improving the selection of existing drugs for further pre-clinical testing and clinical trials for new indications.
